# A New Role for Estrogen Receptor α in Cell Proliferation and Cancer: Activating the Anticipatory Unfolded Protein Response

**DOI:** 10.3389/fendo.2018.00325

**Published:** 2018-06-15

**Authors:** Mara Livezey, Ji Eun Kim, David J. Shapiro

**Affiliations:** ^1^Department of Biochemistry, University of Illinois, Urbana, IL, United States; ^2^Center for Cancer Research, University of Illinois, Urbana, IL, United States

**Keywords:** estrogen, estrogen receptor α, rapid extranuclear signaling, unfolded protein response, calcium, breast cancer, cancer therapy

## Abstract

Cells react to a variety of stresses, including accumulation of unfolded or misfolded protein, by activating the endoplasmic reticulum (EnR) stress sensor, the unfolded protein response (UPR). The UPR is highly conserved and plays a key role in the maintenance of protein folding quality control and homeostasis. In contrast to the classical reactive mode of UPR activation, recent studies describe a hormone-activated anticipatory UPR. In this pathway, mitogenic hormones, such as estrogen (E_2_), epidermal growth factor, and vascular endothelial growth factor rapidly activate the UPR in anticipation of a future need for increased protein folding capacity upon cell proliferation. Here, we focus on this recently unveiled pathway of E_2_-estrogen receptor α (ERα) action. Notably, rapid activation of the anticipatory UPR pathway is essential for subsequent activation of the E_2_-ERα regulated transcription program. Moreover, activation of the UPR at diagnosis is a powerful prognostic marker in ERα positive breast cancer. Furthermore, in cells containing ERα mutations that confer estrogen independence and are common in metastatic breast cancer, the UPR is constitutively activated and linked to antiestrogen resistance. Lethal ERα-dependent hyperactivation of the anticipatory UPR represents a promising therapeutic approach exploited by a new class of small molecule ERα biomodulator.

## Introduction

The endoplasmic reticulum (EnR) plays a key role in the synthesis, folding, and transport of proteins and is important in lipid synthesis (1, 2). Maintenance of protein folding and lipid homeostasis is critical for cell proliferation and viability. The unfolded protein response (UPR) is an EnR stress-response pathway that senses and responds to diverse stimuli, including changes in EnR luminal calcium, redox status, nutrient availability, lipid bilayer composition, and accumulation of unfolded or misfolded protein (3, 4). The UPR consists of three arms, IRE1α, ATF6α, and PERK that together decrease the flux of new protein into the EnR, while simultaneously increasing production of molecular chaperones to help fold unfolded or misfolded proteins. IRE1α and PERK are activated upon oligomerization and autophosphorylation. ATF6α is activated and transported to the Golgi apparatus, where it is cleaved by S1P and S2P proteases, although the mechanism of activation in the EnR is still unclear. There is increasing evidence that all three arms of the UPR can be activated in more than one way. For example, some of the earliest work suggested that the molecular chaperone, BiP, blocked oligomerization, and activation of IRE1α and PERK through direct binding to their luminal domains (2). Upon accumulation of unfolded or misfolded proteins, BiP would be competed away, allowing activation of these UPR arms. Similarly, it is thought that upon depletion of EnR calcium, calcium-dependent molecular chaperones, such as BiP, fall off IRE1α, and PERK, and other unfolded or misfolded proteins. This would allow IRE1α and PERK to oligomerize and activate the UPR (5). Additional experiments and elucidation of the crystal structure of the luminal domain of IRE1α showed that independent of BiP binding, IRE1α can directly bind and be activated by peptides *via* an MHC-like structural domain (2). Interestingly, recent work has also suggested that IRE1α and PERK may sense and be activated by changes in lipid content of the EnR membrane, independent of accumulation of unfolded protein, or depletion of calcium in the EnR (6).

Activation of the non-canonical RNase IRE1α (inositol-requiring enzyme 1α) results in alternative splicing of the transcription factor XBP1, leading to the production of spliced-XBP1 (sp-XBP1) and upregulation of molecular chaperones (7). ATF6α (activating transcription factor 6α) is translocated to the Golgi apparatus where it is cleaved by proteases to produce the transcription factor p50-ATF6α that also upregulates chaperone production (8). Finally, activated PERK (protein kinase RNA-like EnR kinase) phosphorylates eIF2α, resulting in transient inhibition of most protein synthesis, while promoting translation and production of selective proteins, including ATF4, CHOP, p58^IPK^, and GADD34 (9). When UPR stress is mild, the chaperone p58^IPK^ binds to and inhibits PERK, and GADD34 dephosphorylates eIF2α. Working together, p58^IPK^ and GADD34 reverse PERK activation and restore protein synthesis.

Classically, the UPR is activated in response to EnR stress. Several years ago, it was shown that progenitors of immunoglobulin-producing B cells activate the UPR before initiating antibody production. This pathway, which is activated in the absence of unfolded protein, was named the anticipatory UPR by Walter and Ron (2), but it was not studied extensively. We, and others, recently showed that diverse steroid and peptide hormones, including estrogen (17β-estradiol, E_2_), progesterone (P_4_), epidermal growth factor, and vascular endothelial growth factor, and probably the insect hormone ecdysone (Ec), activate an anticipatory UPR pathway to prepare cells for the increased protein folding that accompanies cell proliferation (10–14). Notably, the steps between hormone receptor complexes and activation of the three arms of the UPR have largely been identified (10–12).

The proliferative and anti-apoptotic advantage of overexpressing or activating hormone receptors, such as EGF receptor (EGFR) or estrogen receptor α (ERα), has long been appreciated in cancer biology (15–18). However, hormone activation of the anticipatory UPR has only recently become a focus of cancer research and exploited as a therapeutic target. This review focuses on the role of E_2_-ERα activation of the anticipatory UPR and a promising preclinical drug candidate, BHPI, which leverages this novel action of ERα in order to block proliferation of and kill most ERα positive breast cancer cells.

## Activation of the Anticipatory UPR by Mitogenic Hormones

**Figure 1:**
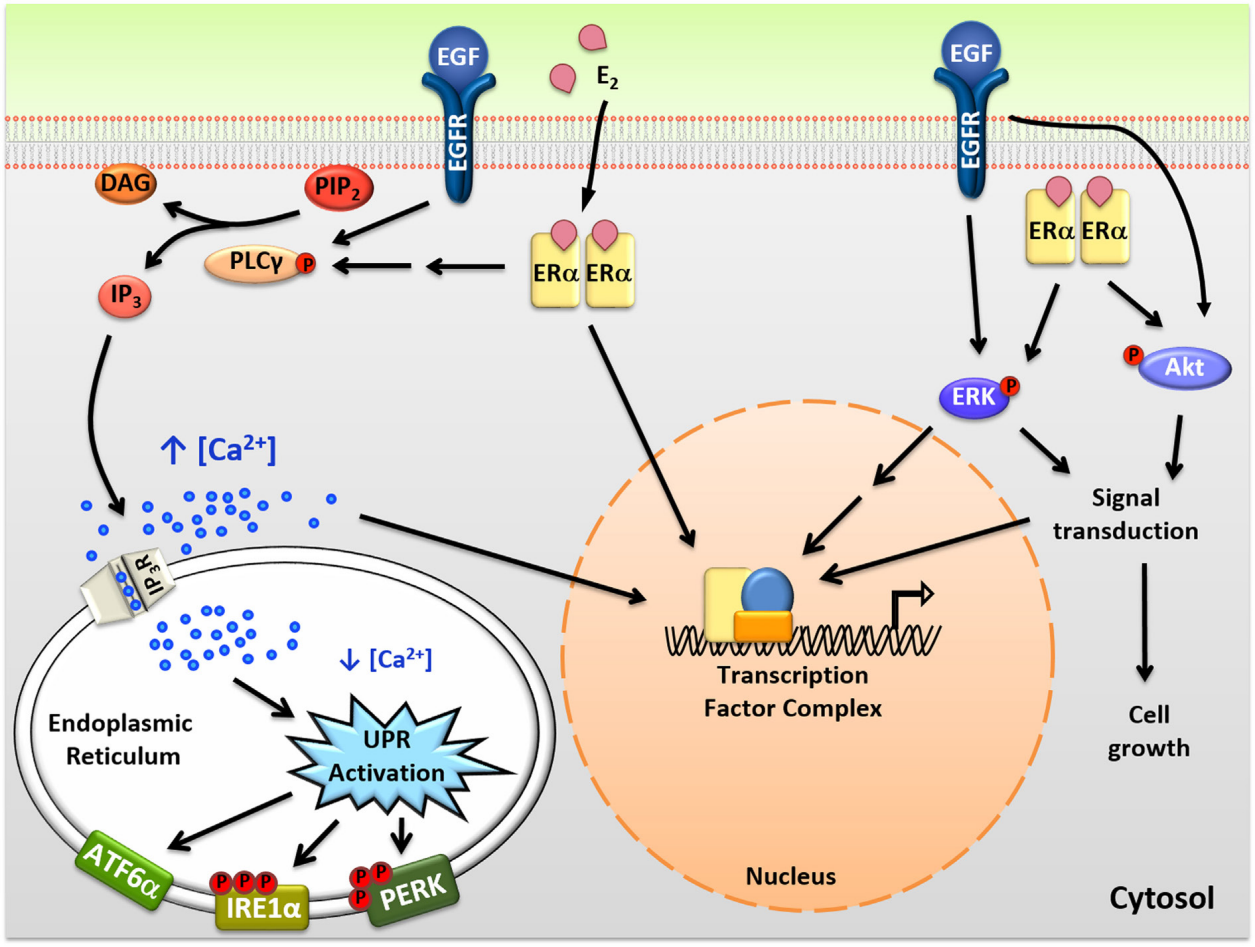
Intracellular actions of mitogenic hormones. Estrogen (E_2_) and epidermal growth factor (EGF) act on their respective receptors, estrogen receptor α (ERα), and EGF receptor (EGFR), to initiate crosstalk between extranuclear signaling pathways and their genomic programs. ERα indirectly and EGFR directly activate phospholipase C γ (PLCγ), resulting in cleavage of PIP_2_ to DAG (diacylglycerol) and IP_3_ (inositol triphosphate). IP_3_ then binds to IP_3_ receptors (IP_3_Rs) in the endoplasmic reticulum (EnR) membrane, causing moderate efflux of calcium from the lumen of the EnR into the cell body. This calcium signal activates all three arms of the unfolded protein response (UPR) and acts as an authorizing signal for E_2_-ERα and EGF-EGFR modulation of gene expression and cell proliferation. In parallel, E_2_-ERα and EGF-EGFR modulate additional extranuclear signal transduction pathways, including activation of ERK and Akt signaling. Activation of these pathways is also important for subsequent cell proliferation and crosstalks with the E_2_-ERα and EGF-EGFR genomic programs.

Steroid and peptide hormones exert their effects through binding and modulating their specific receptors (18, 19). Using E_2_-ERα as an example, when hormone receptors bind to their ligand, they dimerize and are recruited to specific DNA response elements (Figure 1). E_2_-ERα then modulates the activity of thousands of genes either through direct binding to DNA, or by tethering of E_2_-ERα to other transcription factors (20–22). The genomic actions of E_2_-ERα are important for the pro-proliferation properties of E_2_ in ERα positive breast cancer cells and while rapidly initiated, play out over hours or days.

**Figure 2:**
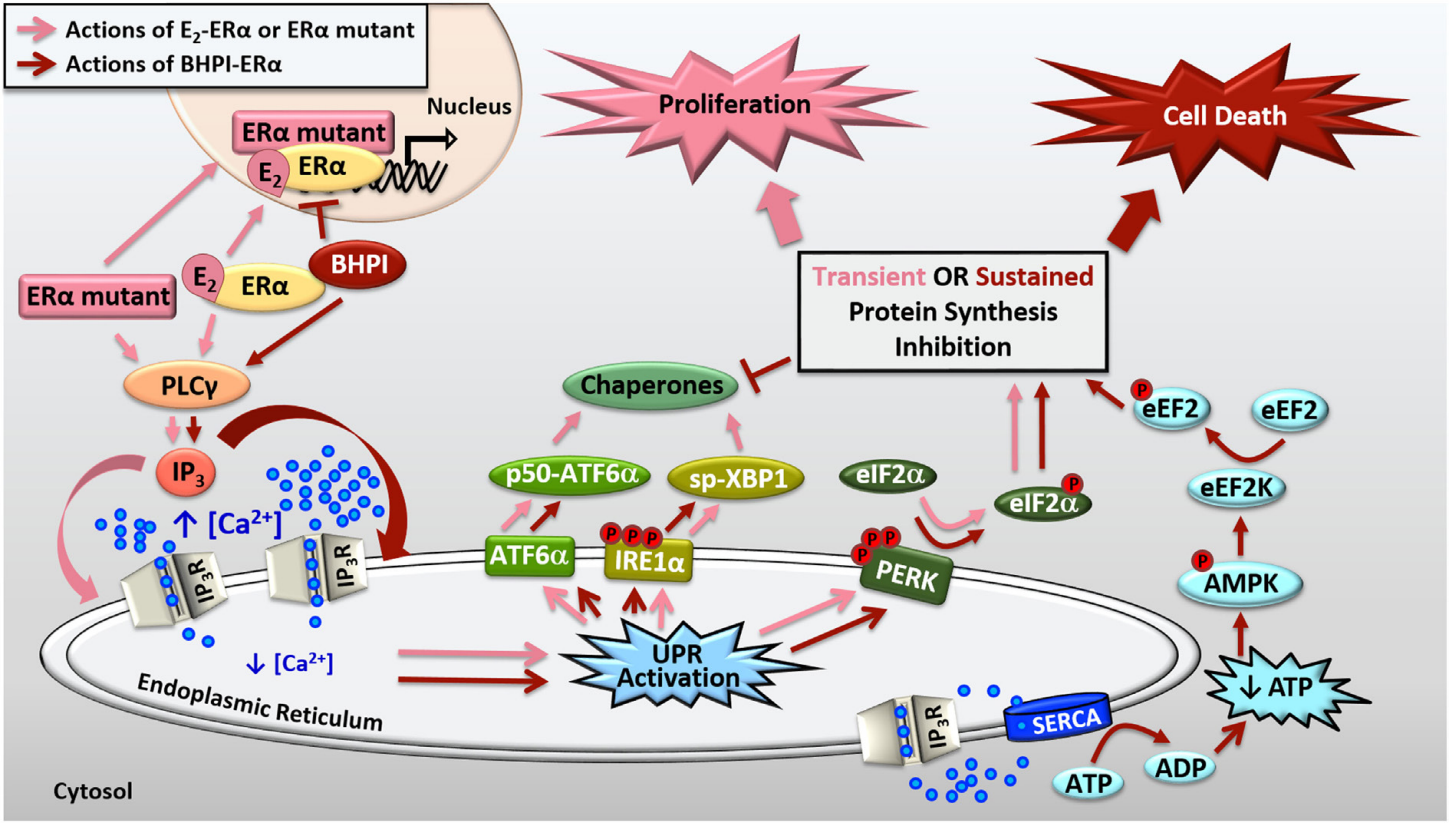
Activation of the anticipatory unfolded protein response by estrogen receptor α (ERα). E_2_-ERα and constitutively active ERα mutants activate a mild and protective anticipatory unfolded protein response (UPR) and the non-competitive biomodulator BHPI binds ERα and induces hyperactivation of this pathway leading to cell death. ERα indirectly activates phospholipase C γ (PLCγ), resulting in cleavage of PIP_2_ to DAG (diacylglycerol) and IP_3_ (inositol triphosphate). E_2_-ERα and constitutively active ERα mutants cause moderate IP_3_ production, whereas BHPI causes significantly more production of IP_3_. The IP_3_ then binds to IP_3_ receptors (IP_3_Rs) in the endoplasmic reticulum (EnR) membrane, causing efflux of calcium from the lumen of the EnR into the cell body. E_2_-ERα and constitutively active ERα mutants cause moderate and transient release of calcium, resulting in weak and transient activation of all three arms of the UPR. Weak UPR activation results in very mild and transient inhibition of protein synthesis, production of molecular chaperones, and is critical for subsequent cell proliferation. BHPI-ERα induced hyperactivation of the UPR causes robust and sustained release of calcium from the EnR. This leads to robust PERK activation and rapid, sustained, and near-quantitative inhibition of protein synthesis. Although BHPI causes upregulation of chaperone mRNA, no protein is made, and the UPR-activating signal is never resolved. In an effort to re-establish cellular calcium homeostasis, ATP-dependent SERCA pumps in the EnR actively transport calcium back into the lumen of the EnR but since IP_3_Rs remain open, an ATP-depleting futile cycle ensues. Decreased cellular ATP and increased AMP activate AMPK, which along with calcium, activates Ca^2+^/calmodulin-dependent kinase, eukaryotic elongation factor 2 kinase (CAMKIII/eEF2K). eEF2K then phosphorylates eEF2, causing inhibition of protein synthesis at elongation. Ultimately, BHPI-ERα induced hyperactivation of the anticipatory UPR causes death of ERα positive endometrial and breast cancer cells.

In addition to classical genomic actions, E_2_-ERα exerts rapid extranuclear actions important for activating signal transduction pathways (Figure 1). These pathways are important for diverse actions of E_2_-ERα, crosstalk with the genomic program, and are rapidly activated and often play out over minutes to hours (23, 24). Of these pathways, activation of the anticipatory UPR is the most recently described (Figure 2) (12). Upon binding of E_2_ to ERα at the plasma membrane, there is rapid activation of phospholipase C γ, resulting in cleavage of its substrate PIP_2_ to IP_3_ (inositol triphosphate) and DAG (diacylglycerol). The IP_3_ then binds to and opens IP_3_ receptor (IP_3_R) calcium channels in the membrane of the EnR, allowing efflux of calcium out of the lumen of the EnR into the cell body. The modest decrease in EnR calcium caused by E_2_ treatment of ERα positive cancer cells weakly activates the UPR, resulting in upregulation of molecular chaperones along with minimal and very transient inhibition of protein synthesis. By knockdown of UPR components, or blocking calcium release from the EnR, we showed that increased intracellular calcium from activation of the anticipatory UPR is critical for subsequent E_2_-ERα-mediated modulation of gene expression and cell proliferation (12).

Consistent with E_2_-ERα activation of the anticipatory UPR, T47D breast cancer cells modified with CRISPR-Cas9 to replace wild-type ERα with the constitutively active mutations ERαY537S (TYS cells) and ERαD538G (TDG cells) upregulate the UPR in the absence of estrogen (13). Strikingly, TYS and TDG cells express higher levels of the molecular chaperones BiP and p58^IPK^ and TYS cells have increased levels of sp-XBP1. Surprisingly and possibly due to robust upregulation of progesterone receptor in these cells lines, P_4_ further elevates some of these downstream products of mild UPR activation, including sp-XBP1, and in TYS cells, p58^IPK^. Of note, the synthetic progestin R5020 increases resistance of TYS and TDG cells to OHT (4-hydroxy tamoxifen; the active form of tamoxifen) and fulvestrant/ICI 182,780 (ICI). In anchorage-independent three-dimensional (3D) assays, R5020-treated TYS and TDG cells exhibited robust proliferation in high concentrations of OHT and fulvestrant/ICI (13).

In diverse cancers, mild UPR activation is protective and helps cancers to proliferate, induce angiogenesis, and overcome hypoxia and toxic stress from chemotherapies (25, 26). Using microarray and outcome data from approximately 1,000 patient breast cancers, we demonstrated the significance of this pathway in ERα positive breast cancer. Increased expression of a UPR gene index consisting of UPR components and UPR-induced chaperones strongly correlated with reduced time to recurrence, subsequent resistance to tamoxifen, and reduced survival (12). The close correlation between the extent of activation of the UPR gene index and activation of E_2_-ERα regulated genes is consistent with ERα playing a major role in the elevated expression of the UPR gene index (10). Moreover, in triple negative breast cancer in which ERα is absent, the IRE1/XBP1 axis plays a central role in tumorigenicity and progression, and the extent of activation of an XBP1 gene index is correlated with reduced patient survival (27, 28). Taken together, this suggests that ERα-mediated activation of the anticipatory UPR likely plays an important role in early survival of breast cancers. At later times when therapeutic stress is added to nutritional deprivation and hypoxia, activation of the classical reactive UPR makes an important contribution to tumor survival (26, 29–31).

Because of the protective nature of the UPR in cancer, drugs that target components of the UPR are in preclinical development, in clinical trials, and have been approved (27, 28, 32). Most commonly, these drugs inhibit key components of the UPR, such as PERK, IRE1α RNase, or the downstream chaperone BiP/GRP78. Unfortunately, due to lack of drug specificity, these drugs may have toxic effects in tissues with a large secretory burden, such as pancreas.

## UPR Hyperactivation as a Tool to Selectively Target ERα Positive Breast Cancer

The standard of care for ERα positive breast cancer is endocrine therapy, including aromatase inhibitors that block E_2_ production, and tamoxifen and fulvestrant/ICI that compete with E_2_ for binding to ERα. Unfortunately, many tumors that were initially responsive recur after years of treatment. Moreover, there is selection and outgrowth of endocrine therapy resistant tumors expressing ERα mutations in about one-third of patients with advanced metastatic breast cancer, most commonly ERαY537S and ERαD538G (33–35). Structural and biophysical studies suggest that estrogen receptors containing these mutations are stabilized in the active conformation and have lower affinity for antiestrogens, such as OHT (36). Additionally, a growing body of clinical evidence suggests that mutations in this ligand binding domain hotspot confer partial resistance to endocrine therapies (33–35, 37). Strikingly, patients whose tumors express ERαY537S or ERαD538G have on average 12 and 6 months shorter survival, respectively, than patients whose tumors express wild-type ERα (38). We have shown that TYS and TDG cells containing these estrogen receptor mutations exhibit constitutively active ERα, allowing E_2_-independent proliferation and gene expression, and partial resistance to the endocrine therapies OHT and fulvestrant/ICI (13). Additionally, compared to wild-type ERα in T47D cells, we observed resistance to fulvestrant/ICI-mediated degradation of the mutant ERαs in TYS and TDG cells. Because of the resistance to endocrine therapies observed in cancers expressing ERαY537S and ERαD538G, development of better selective estrogen receptor modulators and degraders (SERMs and SERDs) has been a focus in targeting these cancers (39–42).

Recently, we described a novel small molecule biomodulator, BHPI, that selectively targets ERα positive cancer cells (13, 43, 44). BHPI[3,3-bis(4-hydroxyphenyl)-7-methyl-1,3,dihydro-2H-indol-2-one] is a bis-phenylated oxoindol. In a limited structure-activity-relationship study, addition of methyl groups to both phenyl rings significantly disrupted activity of BHPI (43). We demonstrated specificity by testing over 30 ERα positive and negative cell lines and showed that BHPI only inhibits proliferation of or kills cells that express ERα (43). Additionally, in the isogenic human breast cell lines MCF10A (ERα negative) and MCF10A_ER IN9_ (ERα positive), we showed that BHPI was only effective in the cells expressing ERα, and was ineffective when ERα was knocked back down in MCF10A_ER IN9_ cells. Demonstrating that BHPI physically interacts with ERα, BHPI shifts the tryptophan emission spectrum of ERα, and protects peptides in the ERα ligand binding domain from protease digestion. Furthermore, BHPI inhibits recruitment of ERα to E_2_-ERα regulated promoters (Figure 2). However, BHPI is not a competitive inhibitor, as it does not compete with radiolabeled E_2_ for the ligand binding pocket and is equally effective in the presence and absence of estrogen (43). Rather than inhibiting a component of the UPR, BHPI takes advantage of the already moderately elevated UPR in cancer cells by hyperactivating the anticipatory UPR (Figure 2). BHPI, therefore, hijacks the normal protective actions of ERα activation of the UPR in order to push UPR activation into the lethal range. This is the first small molecule to modulate the action of a hormone receptor in this way.

We showed that BHPI blocks proliferation of ovarian cancer cells and kills most ERα positive breast and endometrial cancer cells (13, 43, 45). Compared to E_2_, BHPI causes increased production of IP_3_ in cancer cell lines (43, 45). This increased production of IP_3_ hyperactivates the UPR through sustained opening of IP_3_R calcium channels in the EnR, resulting in robust and sustained calcium release from the lumen of the EnR into the cell body (Figure 2). While E_2_ causes mild and transient inhibition of protein synthesis, BHPI causes a rapid, sustained, and near-quantitative inhibition of protein synthesis in ERα positive breast and endometrial cancer cells. Surprisingly, BHPI also causes rapid depletion of intracellular ATP. Disruption of cytosolic calcium homeostasis can be toxic, specifically, high levels of calcium can lead to cell death (45–47). To restore intracellular calcium homeostasis after opening of IP_3_Rs, ATP-dependent SERCA pumps calcium back into the EnR lumen, but because IP_3_Rs remain open, the calcium leaks back out. This creates a futile cycle of calcium leakage and pumping that depletes intracellular ATP. Additionally, ATP depletion from prolonged SERCA pump activity results in increased levels of cellular AMP that activates the metabolic sensor AMPK. AMPK activation together with high levels of cytosolic calcium activates the Ca^2+^/calmodulin-dependent kinase, eukaryotic elongation factor 2 kinase (CAMKIII/eEF2K). eEF2K then phosphorylates eEF2, which inhibits protein synthesis at a second site (Figure 2). Therefore, although BHPI increases the mRNA levels of chaperones, such as BiP and p58^IPK^, no protein is made, leading to un-resolvable cytotoxic activation of the UPR. While other activators of the classical reactive UPR share similarities to BHPI’s mechanism of action, such as disruption of EnR calcium homeostasis and inhibition of protein synthesis (1, 2), BHPI is unique in its ability to cause ATP depletion in cancer cells.

We recently described the efficacy of targeting breast cancer cells expressing ERαY537S and ERαD538G with BHPI (13). In 3D culture, OHT and fulvestrant/ICI only partially inhibited growth of TYS and TDG cells and R5020 completely reversed antiestrogen inhibition of growth. In contrast, BHPI killed TYS and TDG cells in the presence or absence of R5020. Since BHPI is not a competitive inhibitor of ERα (43) and targets ERα positive cancer cells irrespective of their dependence on E_2_ for proliferation, it is a promising preclinical drug candidate for the treatment of metastatic breast cancers expressing ERαY537S and ERαD538G.

In ovarian cancer, a common mechanism for resistance to the taxane paclitaxel and other chemotherapy agents is overexpression of ATP-dependent efflux pumps, especially Multidrug Resistance Protein 1 (MDR1)/P-glycoprotein/ABCB1. Despite intensive efforts, effective and non-toxic MDR1 inhibitors have remained elusive. Due to its ability to deplete intracellular ATP, BHPI inhibited ATP-dependent MDR1-mediated drug efflux and restored sensitivity of multidrug-resistant breast and ovarian cancer cells to killing by therapeutically relevant concentrations of several anticancer drugs (44). Using multidrug resistant OVCAR-3 cells, BHPI was tested in an orthotopic ovarian cancer xenograft model. Although paclitaxel was ineffective against these tumors, BHPI alone strongly reduced tumor growth. Notably, tumors were undetectable in mice treated with BHPI plus paclitaxel. After the combination therapy, plasma levels of the widely used cancer biomarker, CA125, were at least several hundred fold lower than in mice with control tumors. Moreover, CA125 levels progressively declined to undetectable in all mice treated with the combination therapy (44).

## Conclusion

Studies of the pro-proliferative effects of mitogenic hormones and their respective receptors have long focused on their actions on genomic programs and on extranuclear signal transduction pathways. Activation of the anticipatory UPR is an emerging, very rapid action of many mitogenic hormones that authorizes subsequent gene expression and cell proliferation. Important for targeting hormone receptor positive breast cancers is the finding that they exhibit elevated UPR activation. This UPR activation can be exploited by small molecules that hyperactivate the pathway, pushing UPR activation into the lethal range. As a first-in-class small molecule, BHPI is a model for investigating hyperactivation of the anticipatory UPR as a promising strategy for killing ERα positive breast cancer cells. A similar approach is also likely viable for breast cancers that overexpress other hormone receptors that activate the anticipatory UPR, such as progesterone receptor, or EGFR family members. However, agents that hyperactivate the anticipatory UPR through these receptors have yet to be identified. Thus, the anticipatory UPR is a key pathway for development of new anticancer drugs that can help overcome resistance to current therapies.

## Author Contributions

ML and DS contributed to writing and revising. JK contributed to revising the manuscript.

## Conflict of Interest Statement

The authors declare that the research was conducted in the absence of any commercial or financial relationships that could be construed as a potential conflict of interest.

## Abbreviations

EnR, endoplasmic reticulum; TYS or TDG, T47D cells expressing either ERαY537S or ERαD538G.

## References

[1] Ron D Walter P. Signal integration in the endoplasmic reticulum unfolded protein response. Nat Rev Mol Cell Biol (2007) 8:519–29. 10.1038/nrm2199 17565364

[2] Walter P Ron D. The unfolded protein response: from stress pathway to homeostatic regulation. Science (2011) 334:1081–6. 10.1126/science.1209038 22116877

[3] Urra H Dufey E Avril T Chevet E Hetz C. Endoplasmic reticulum stress and the hallmarks of cancer. Trends Cancer (2016) 2:252–62. 10.1016/j.trecan.2016.03.007 28741511

[4] Korennykh A Walter P. Structural basis of the unfolded protein response. Annu Rev Cell Dev Biol (2012) 28:251–77. 10.1146/annurev-cellbio-101011-155826 23057742

[5] Preissler S Chambers JE Crespillo-Casado A Avezov E Miranda E Perez J Physiological modulation of BiP activity by trans-protomer engagement of the interdomain linker. Elife (2015) 4:e08961. 10.7554/eLife.08961 26473973 PMC4608358

[6] Volmer R Ron D. Lipid-dependent regulation of the unfolded protein response. Curr Opin Cell Biol (2015) 33:67–73. 10.1016/j.ceb.2014.12.002 25543896 PMC4376399

[7] Cox JS Walter P. A novel mechanism for regulating activity of a transcription factor that controls the unfolded protein response. Cell (1996) 87:391–404. 10.1016/S0092-8674(00)81360-4 8898193

[8] Yoshida H Haze K Yanagi H Yura T Mori K. Identification of the cis-acting endoplasmic reticulum stress response element responsible for transcriptional induction of mammalian glucose-regulated proteins. Involvement of basic leucine zipper transcription factors. J Biol Chem (1998) 273:33741–9. 10.1074/jbc.273.50.33741 9837962

[9] Novoa I Zeng H Harding HP Ron D Feedback inhibition of the unfolded protein response by GADD34-mediated dephosphorylation of eIF2α. J Cell Biol (2001) 153:1011–22. 10.1083/jcb.153.5.1011 11381086 PMC2174339

[10] Karali E Bellou S Stellas D Klinakis A Murphy C Fotsis T. VEGF signals through ATF6 and PERK to promote endothelial cell survival and angiogenesis in the absence of ER stress. Mol Cell (2014) 54:559–72. 10.1016/j.molcel.2014.03.022 24746698

[11] Yu L Andruska N Zheng X Shapiro DJ. Anticipatory activation of the unfolded protein response by epidermal growth factor is required for immediate early gene expression and cell proliferation. Mol Cell Endocrinol (2016) 422:31–41. 10.1016/j.mce.2015.11.005 26551735 PMC4919024

[12] Andruska N Zheng X Yang X Helferich WG Shapiro DJ. Anticipatory estrogen activation of the unfolded protein response is linked to cell proliferation and poor survival in estrogen receptor α-positive breast cancer. Oncogene (2015) 34:3760–9. 10.1038/onc.2014.292 25263449 PMC4377305

[13] Mao C Livezey M Kim JE Shapiro DJ. Antiestrogen resistant cell lines expressing estrogen receptor α mutations upregulate the unfolded protein response and are killed by BHPI. Sci Rep (2016) 6:34753. 10.1038/srep34753 27713477 PMC5054422

[14] Liu W Cai M-J Zheng C-C Wang J-X Zhao X-F. Phospholipase Cγ1 connects the cell membrane pathway to the nuclear receptor pathway in insect steroid hormone signaling. J Biol Chem (2014) 289:13026–41. 10.1074/jbc.M113.547018 24692553 PMC4036317

[15] Normanno N De Luca A Bianco C Strizzi L Mancino M Maiello MR Epidermal growth factor receptor (EGFR) signaling in cancer. Gene (2006) 366:2–16. 10.1016/j.gene.2005.10.018 16377102

[16] Deroo BJ Korach KS Estrogen receptors and human disease. J Clin Invest (2006) 116:561–70. 10.1172/JCI27987 16511588 PMC2373424

[17] Evans RM. The steroid and thyroid hormone receptor superfamily. Science (1988) 240:889–95. 10.1126/science.3283939 3283939 PMC6159881

[18] Huang P Chandra V Rastinejad F. Structural overview of the nuclear receptor superfamily: insights into physiology and therapeutics. Annu Rev Physiol (2010) 72:247–72. 10.1146/annurev-physiol-021909-135917 20148675 PMC3677810

[19] Katzenellenbogen BS Dynamics of steroid hormone receptor action. Annu Rev Physiol (1980) 42:17–35. 10.1146/annurev.ph.42.030180.000313 6996577

[20] York B O’Malley BW. Steroid receptor coactivator (SRC) family: masters of systems biology. J Biol Chem (2010) 285:38743–50. 10.1074/jbc.R110.193367 20956538 PMC2998129

[21] Carroll JS Liu XS Brodsky AS Li W Meyer CA Szary AJ Chromosome-wide mapping of estrogen receptor binding reveals long-range regulation requiring the forkhead protein FoxA1. Cell (2005) 122:33–43. 10.1016/j.cell.2005.05.008 16009131

[22] Hah N Danko CG Core L Waterfall JJ Siepel A Lis JT A rapid, extensive, and transient transcriptional response to estrogen signaling in breast cancer cells. Cell (2011) 145:622–34. 10.1016/j.cell.2011.03.042 21549415 PMC3099127

[23] Levin ER Plasma membrane estrogen receptors. Trends Endocrinol Metab (2009) 20:477–82. 10.1016/j.tem.2009.06.009 19783454 PMC3589572

[24] Song RX-D Santen RJ Membrane initiated estrogen signaling in breast cancer. Biol Reprod (2006) 75:9–16. 10.1095/biolreprod.105.050070 16571873

[25] Ma Y Hendershot LM. The role of the unfolded protein response in tumour development: friend or foe? Nat Rev Cancer (2004) 4:966–77. 10.1038/nrc1505 15573118

[26] Wang M Kaufman RJ. The impact of the endoplasmic reticulum protein-folding environment on cancer development. Nat Rev Cancer (2014) 14:581–97. 10.1038/nrc3800 25145482

[27] Chen X Iliopoulos D Zhang Q Tang Q Greenblatt MB Hatziapostolou M XBP1 promotes triple-negative breast cancer by controlling the HIF1α pathway. Nature (2014) 508:103–7. 10.1038/nature13119 24670641 PMC4105133

[28] Zhao N Cao J Xu L Tang Q Dobrolecki LE Lv X Pharmacological targeting of MYC-regulated IRE1/XBP1 pathway suppresses MYC-driven breast cancer. J Clin Invest (2018) 128:1283–99. 10.1172/JCI95873 29480818 PMC5873887

[29] Wang M Kaufman RJ. Protein misfolding in the endoplasmic reticulum as a conduit to human disease. Nature (2016) 529:326–35. 10.1038/nature17041 26791723

[30] Clarke R Cook KL Hu R Facey COB Tavassoly I Schwartz JL Endoplasmic reticulum stress, the unfolded protein response, autophagy, and the integrated regulation of breast cancer cell fate. Cancer Res (2012) 72:1321–31. 10.1158/0008-5472.CAN-11-3213 22422988 PMC3313080

[31] Rajapaksa G Thomas C Gustafsson J-Å. Estrogen signaling and unfolded protein response in breast cancer. J Steroid Biochem Mol Biol (2016) 163:45–50. 10.1016/j.jsbmb.2016.03.036 27045680

[32] Sykes EK Mactier S Christopherson RI. Melanoma and the unfolded protein response. Cancers (2016) 8:E30. 10.3390/cancers8030030 26927180 PMC4810114

[33] Robinson DR Wu Y-M Vats P Su F Lonigro RJ Cao X Activating ESR1 mutations in hormone-resistant metastatic breast cancer. Nat Genet (2013) 45:1446–51. 10.1038/ng.2823 24185510 PMC4009946

[34] Toy W Shen Y Won H Green B Sakr RA Will M ESR1 ligand-binding domain mutations in hormone-resistant breast cancer. Nat Genet (2013) 45:1439–45. 10.1038/ng.2822 24185512 PMC3903423

[35] Spoerke JM Gendreau S Walter K Qiu J Wilson TR Savage H Heterogeneity and clinical significance of ESR1 mutations in ER-positive metastatic breast cancer patients receiving fulvestrant. Nat Commun (2016) 7:11579. 10.1038/ncomms11579 27174596 PMC4869259

[36] Fanning SW Mayne CG Dharmarajan V Carlson KE Martin TA Novick SJ Estrogen receptor alpha somatic mutations Y537S and D538G confer breast cancer endocrine resistance by stabilizing the activating function-2 binding conformation. Elife (2016) 5:e12792. 10.7554/eLife.12792 26836308 PMC4821807

[37] Merenbakh-Lamin K Ben-Baruch N Yeheskel A Dvir A Soussan-Gutman L Jeselsohn R D538G mutation in estrogen receptor-α: a novel mechanism for acquired endocrine resistance in breast cancer. Cancer Res (2013) 73:6856–64. 10.1158/0008-5472.CAN-13-1197 24217577

[38] Chandarlapaty S Chen D He W Sung P Samoila A You D Prevalence of ESR1 mutations in cell-free DNA and outcomes in metastatic breast cancer: a secondary analysis of the BOLERO-2 clinical trial. JAMA Oncol (2016) 2:1310–5. 10.1001/jamaoncol.2016.1279 27532364 PMC5063698

[39] Zhao Y Laws MJ Guillen VS Ziegler Y Min J Sharma A Structurally novel antiestrogens elicit differential responses from constitutively active mutant estrogen receptors in breast cancer cells and tumors. Cancer Res (2017) 77:5602–13. 10.1158/0008-5472.CAN-17-1265 28904064 PMC5645250

[40] Weir HM Bradbury RH Lawson M Rabow AA Buttar D Callis RJ AZD9496: an oral estrogen receptor inhibitor that blocks the growth of ER-positive and ESR1-mutant breast tumors in preclinical models. Cancer Res (2016) 76:3307–18. 10.1158/0008-5472.CAN-15-2357 27020862

[41] Xiong R Zhao J Gutgesell LM Wang Y Lee S Karumudi B Novel selective estrogen receptor downregulators (SERDs) developed against treatment-resistant breast cancer. J Med Chem (2017) 60:1325–42. 10.1021/acs.jmedchem.6b01355 28117994 PMC5786431

[42] Bihani T Patel HK Arlt H Tao N Jiang H Brown JL Elacestrant (RAD1901), a selective estrogen receptor degrader (SERD), has antitumor activity in multiple ER+breast cancer patient-derived xenograft models. Clin Cancer Res (2017) 23:4793–804. 10.1158/1078-0432.CCR-16-2561 28473534

[43] Andruska ND Zheng X Yang X Mao C Cherian MM Mahapatra L Estrogen receptor α inhibitor activates the unfolded protein response, blocks protein synthesis, and induces tumor regression. Proc Natl Acad Sci U S A (2015) 112:4737–42. 10.1073/pnas.1403685112 25825714 PMC4403155

[44] Zheng X Andruska N Lambrecht MJ He S Parissenti A Hergenrother PJ Targeting multidrug-resistant ovarian cancer through estrogen receptor α dependent ATP depletion caused by hyperactivation of the unfolded protein response. Oncotarget (2018) 9:14741–53. 10.18632/oncotarget.10819 29599904 PMC5871075

[45] Berridge MJ Bootman MD Roderick HL. Calcium signalling: dynamics, homeostasis and remodelling. Nat Rev Mol Cell Biol (2003) 4:517–29. 10.1038/nrm1155 12838335

[46] Criddle DN Gerasimenko JV Baumgartner HK Jaffar M Voronina S Sutton R Calcium signalling and pancreatic cell death: apoptosis or necrosis? Cell Death Differ (2007) 14:1285–94. 10.1038/sj.cdd.4402150 17431416

[47] Bruce JIE. Metabolic regulation of the PMCA: role in cell death and survival. Cell Calcium (2018) 69:28–36. 10.1016/j.ceca.2017.06.001 28625348 PMC5761718

